# Histone chaperone FACT is essential to overcome replication stress in mammalian cells

**DOI:** 10.1038/s41388-020-1346-9

**Published:** 2020-06-12

**Authors:** Laura Prendergast, Erin Hong, Alfiya Safina, Dante Poe, Katerina Gurova

**Affiliations:** Department of Cell Stress Biology, Roswell Park Comprehensive Cancer Center, Buffalo, NY 14263 USA

**Keywords:** Breast cancer, DNA replication

## Abstract

The histone chaperone FACT is upregulated during mammary tumorigenesis and necessary for the viability and growth of breast tumor cells. We established that only proliferating tumor cells are sensitive to FACT knockdown, suggesting that FACT functions during DNA replication in tumor cells but not in normal cells. We hypothesized that the basal level of replication stress defines the FACT dependence of cells. Using genetic and chemical tools, we demonstrated that FACT is needed to overcome replication stress. In the absence of FACT during replication stress, the MCM2-7 helicase dissociates from chromatin, resulting in the absence of ssDNA accumulation, RPA binding, and activation of the ATR/CHK1 checkpoint response. Without this response, stalled replication forks are not stabilized, and new origin firing cannot be prevented, leading to the accumulation of DNA damage and cell death. Thus, we propose a novel role for FACT as a factor preventing helicase dissociation from chromatin during replication stress.

## Introduction

The histone chaperone facilitates chromatin transcription (FACT) is involved in multiple chromatin-related processes, including transcription, replication, and DNA repair (reviewed in [1]). It is conserved in all eukaryotes. While it is essential for the viability of yeast cells, multiple cells in other organisms can survive and even grow in the absence of FACT [2–8]. The processes or mechanisms that make cells dependent on FACT and the functions of FACT in these processes are still unclear.

FACT is overexpressed in multiple cancer types, including breast cancer, in which its expression correlates with known markers of poor prognosis, decreased survival, and a high probability of metastatic disease [3, 4, 9–11]. The viability and growth of breast cancer cells, but not normal cells, are dependent on FACT, suggesting that FACT may be a promising anticancer target [3, 4]. The most well-characterized function of FACT is to assist RNA polymerase elongation through chromatin. However, FACT knockdown in a panel of breast cancer cells demonstrated that even cells that are the most sensitive to FACT depletion have no change in their general transcription rates [3]. Moreover, FACT knockdown in breast cancer cells caused changes in the expression of a very small number of genes, which had little overlap between cell lines and no FACT enrichment [3]. Little difference in the expression of FACT-enriched genes was also recently reported in different organisms [7, 12, 13].

Besides transcription elongation, several reports demonstrated FACT involvement in DNA replication. In Saccharomyces cerevisiae, both FACT subunits were co-purified with DNA polymerase-α and required for replication [14, 15]. In mouse cells, FACT co-localized with replication foci during S-phase [16]. Proteomic analysis of human tumor cell extracts showed that both FACT subunits could interact with the MCM2-7 helicase through the MCM4 subunit. In addition, Tan et al. [17] found that FACT is essential for helicase activity on a nucleosomal template, but not on naked DNA, in cell-free conditions and is required for initiation and elongation of replication in HeLa cells. Moreover, SSRP1-depleted transformed chicken DT40 cells did not grow and had a delay in S-phase cell cycle progression; however, SSRP1 depletion did not affect the level of chromatin-bound DNA polymerase-α or nucleosome reassembly on the daughter strands [18]. All these studies suggest that FACT plays an essential conserved role during DNA replication. At the same time, several studies using non-tumor mammalian cells found no difference in the proliferation of primary mammary epithelial cells and mouse fibroblasts in the presence or absence of FACT, and proliferation of mouse embryonic stem cells (ESC) was even increased in the absence of FACT [4, 7, 19]. Thus, not all cells require FACT for replication, or FACT may play a more specific role in DNA replication that is vital for tumor cells, but not normal cells.

In this study, we investigated why the replication of non-tumor mammalian cells may occur in the absence of FACT and what makes tumor cell replication dependent on FACT. We tested and confirmed the hypothesis that FACT is crucial for cells with high levels of replication stress and demonstrated the mechanism of how FACT supports replication stress-specific checkpoint signaling. While tumor cells with a high basal level of replication stress accumulate DNA damage and die in the absence of FACT-mediated checkpoint control, normal cells with a low basal level of replication stress avoid this damage and cell death, even in the absence of FACT. Recent studies have demonstrated that compromising replication stress-responsive checkpoints in cancer cells may provide a novel therapeutic approach to target tumor cells with high endogenous levels of replication stress with minimal effects on normal cells [20].

## Results

### FACT is essential for viability of proliferating tumor cells, but not for growth-arrested transformed cells or proliferating non-tumor cells

**Fig. 1:**
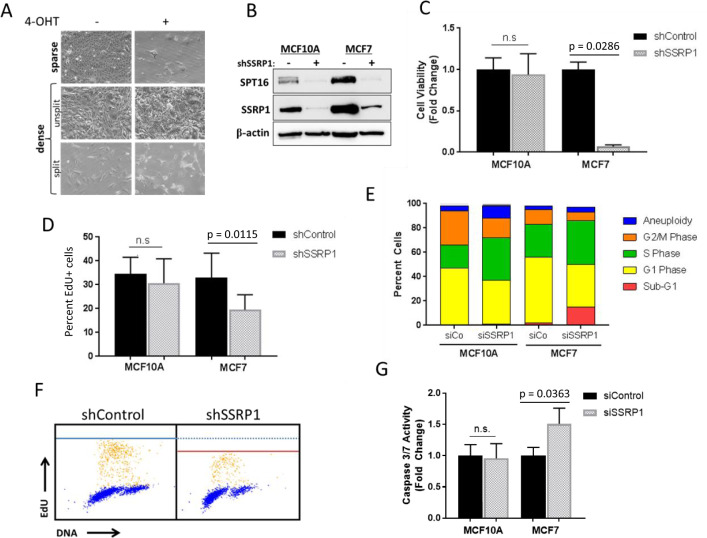
FACT is essential for the viability of proliferating tumor cells, but not of growth-arrested transformed cells or growing non-tumor cells. **a** The effects of *Ssrp1* KO on growing and growth-arrested transformed mouse fibroblasts from *Ssrp1*^*fl/fl*^; CreER^T2+/+^ mice. Microphotographs of cells plated at two different densities, sparse (~10% confluency) in 10% FBS or dense (100% confluency) in 1% FBS, and grown in the presence or absence of 4-OHT for 5 days. Images were taken at the end of 4-OHT treatment for sparse cells and 10 days posttreatment for dense cells, which were either split or not 3 days before imaging. **b**–**g** Human mammary epithelial cells, immortalized MCF10A or tumor MCF7, were evaluated 72 h after transduction with lentiviral shRNAs or transfection with siRNAs as indicated, except **c**. **b** SSRP1 knockdown (KD) in MCF10A and MCF7 cells. Immunoblotting of cell lysates (“−” shControl). **c** Effect of SSRP1 KD on MCF10A and MCF7 cells viability. Cells were sparsely seeded 72 h post-transduction, allowed to grow for 10 days, and then stained with methylene blue. Data are presented as the mean ± SD (*n* = 3) normalized to the corresponding shControl. **d** Effect of SSRP1 KD on DNA replication of MCF10A and MCF7 cells. EdU incorporation was quantified using immunofluorescence imaging. Data are presented as the mean ± SD (*n* = 3). **e** Quantitation of cell cycle distribution using flow cytometry. **f** Cell cycle analysis using co-staining with EdU and Hoechst (yellow dots, EdU+ cells; blue dots, EdU− cells). Blue and red lines represent the maximum intensity of EdU in shControl and shSSRP1, respectively. **g** Caspase-3 and -7 activity. Data are presented as the mean ± SD (*n* = 3).

To determine the role of DNA replication in the sensitivity of tumor cells to FACT loss, we first attempted to compare the toxicity of FACT knockdown between proliferating and growth-arrested cells. However, we were unable to arrest breast cancer cells that were sensitive to FACT depletion. Thus, we utilized transformed mouse fibroblasts from *Ssrp1*^*fl/fl*^; CreER^T2+/+^ mice, which die upon *Ssrp1* deletion [8, 21]. Unlike the breast cancer cells, these cells could be growth arrested by contact inhibition and serum withdrawal. Proliferating or quiescent (high density, low serum) cells were treated with 4-hydroxytamoxifen (4-OHT) to induce *Ssrp1* excision (Fig. S1A). *Ssrp1* KO caused a severe decrease in the viability of the proliferating cells, but not the quiescent cells (Fig. 1a). Moreover, although the growth-arrested *Ssrp1* KO cells lived for an extended time (2 weeks), they died shortly after re-plating at low density in normal serum (i.e., within 3–4 days) (Fig. 1a). These data suggested that FACT is essential for maintaining the viability of proliferating, but not growth-arrested, transformed cells.

To understand why the proliferation of tumor cells, but not normal breast cells, depends on FACT, we utilized MCF10A cells, which represent a model of immortalized, non-tumorigenic, mammary epithelial cells. These cells exhibit many features of normal breast epithelium, including lack of anchorage-independent growth, sensitivity to contact inhibition, and dependence on growth factors and hormones for proliferation and survival [22]. These cells express moderate protein levels of both FACT subunits (SSRP1 and SPT16), which are significantly lower than the levels observed in breast cancer cells (e.g., MCF7) (Fig. 1b). To deplete FACT from cells in this study, we used knockdown of the SSRP1 subunit with either shRNA (for longer experiments) or siRNA (for short-term experiments) to different regions of SSRP1. Both subunits are stable only when they are bound to each other, and, therefore, knockdown of one subunit is sufficient to eliminate the whole complex [23]. Scrambled shRNA and siRNA were used as controls (shControl and siControl, respectively).

We compared the viability of MCF10A and MCF7 cells upon FACT knockdown. While MCF7 cells did not grow without FACT, there was no significant difference in the growth of shSSRP1 and shControl MCF10A cells (Fig. 1c). Consistent with this result, there was a significant reduction in DNA replication in the MCF7 cells 72 h post-transduction with SSRP1 shRNA, but no significant difference in DNA replication between the control and FACT-depleted MCF10A cells (Fig. 1d).

Despite the lack of changes in the viability and DNA replication of MCF10A cells following FACT knockdown, the cell cycle distribution of these FACT-depleted cells changed, including the accumulation of cells in S-phase, a reduction in the number of cells in the G2/M phase, and a slight increase in aneuploidy (Figs. 1e and S1B). Moreover, two-dimensional cell cycle analysis [24, 25] showed that cells depleted of FACT accumulated less EdU per cell during the same period of time compared with the control cells, suggesting that they progressed through the S-phase at a slower rate (Fig. 1f). MCF7 cells depleted of FACT also had an increased percentage of cells in S-phase, which was accompanied by a large accumulation of cells in sub-G1, suggesting that the loss of FACT caused S-phase defects and cell death (Figs. 1e and S1B). These results were consistent with the increased caspase-3/7 activity observed in MCF7 cells, but not MCF10A cells, upon FACT depletion (Fig. 1g). Taken together, these data show that breast cancer cells cannot properly replicate in the absence of FACT and subsequently die, whereas non-tumor mammary epithelial cells continue to replicate DNA at a slower rate.

### Dysregulation of replication renders mammary epithelial cells more sensitive to FACT knockdown

**Fig. 2:**
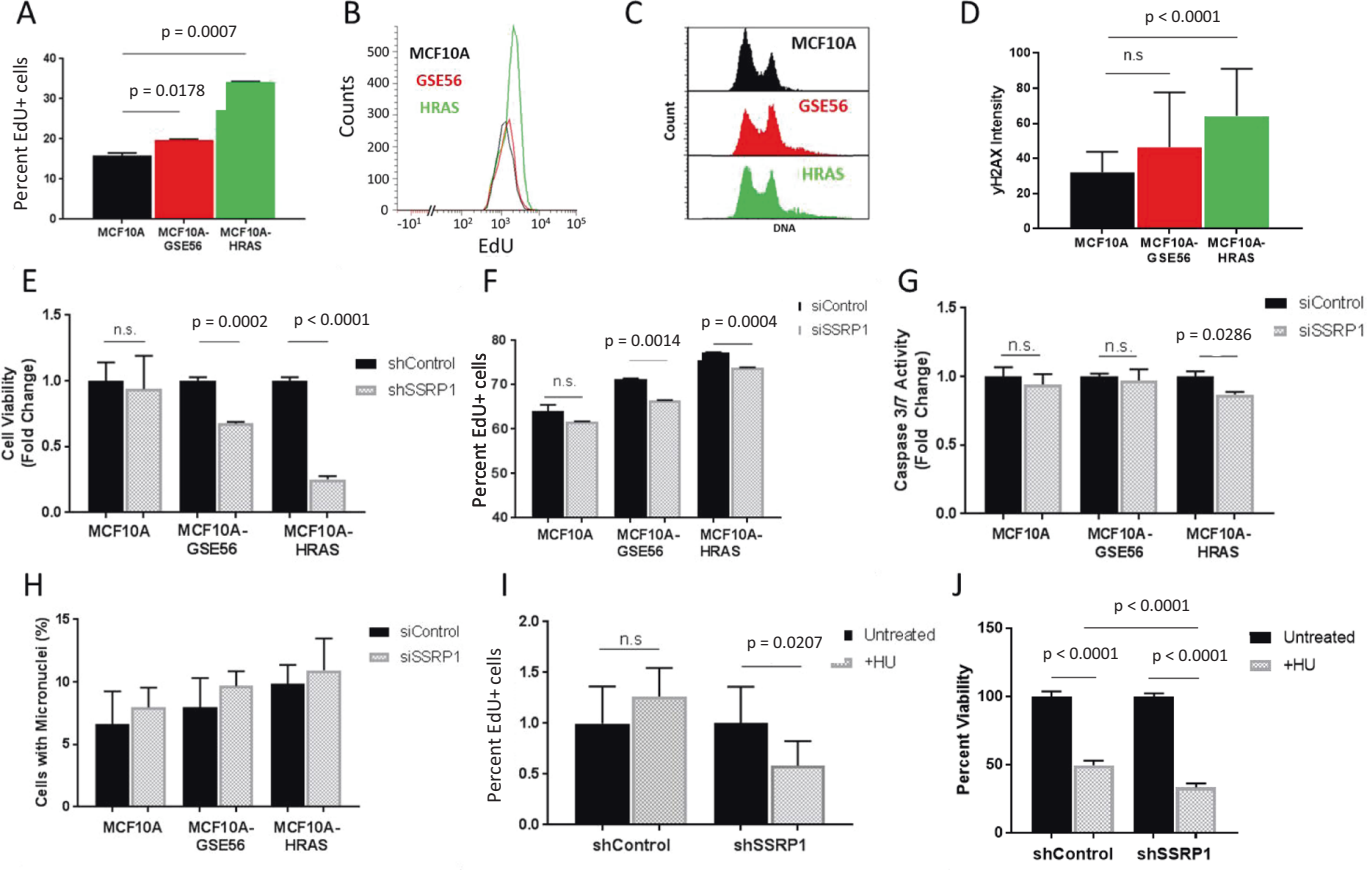
Dysregulation of replication renders mammary epithelial cells more sensitive to FACT knockdown. **a**, **b** Inactivation of p53 and overexpression of HRAS^V12^ increases MCF10A replication. EdU incorporation was analyzed using flow cytometry. **a** The percentage of EdU+ cells in the cell populations. Data are presented as the mean ± SD (*n* = 2). **b** Histograms of EdU intensity. **c** Cell cycle distribution of PI-stained cells measured via flow cytometry. **d** Immunofluorescence staining of γ-H2AX. The γ-H2AX intensity was measured via flow cytometry and presented as the area under the γ-H2AX curve. **e** Cell viability was measured by methylene blue staining 10 days following sparse plating after transduction. Data are presented as the mean ± SD (*n* = 3) normalized to the shControl. **f** EdU incorporation was measured 72 h post-transfection with control or SSRP1 siRNAs. EdU+ cells were quantified using immunofluorescence imaging. Data are presented as the mean ± SD (*n* = 3). **g** Caspase-3/7 activity was measured 72 h post-transfection of cells with control or SSRP1 siRNAs. Data are presented as the mean ± SD (*n* = 3). **h** Micronuclei were quantified by staining with Hoechst. Data are presented as the mean ± SD (*n* = 3). **i**, **j** MCF10A cells were transduced with control or SSRP1 shRNA lentivirus and then incubated in the presence or absence of 1 mM HU for 12 h. **i** EdU incorporation was measured 24 h after the removal of HU. EdU+ cells were quantified using immunofluorescence imaging. Data are presented as the mean ± SD (*n* = 3). **j** After the removal of HU, cells were grown for 8 days. The percent viability was quantified using the methylene blue assay. Data are presented as the mean ± SD (*n* = 3).

In cancer, oncogenic mutations in growth regulators or loss of tumor suppressors frequently lead to dysregulation of DNA replication. To model these processes, we transduced MCF10A cells with either mutant oncogene HRAS^V12^ or a dominant inhibitor of the tumor suppressor p53 (GSE56) [26]. Overexpression of HRAS^V12^ in MCF10A-HRAS cells was confirmed by western blotting (Fig. S2A), and functional inhibition of p53 in MCF10A-GSE56 cells was established by the loss of the p53 response to the p53-activating compound, CBL0137 [27] (Fig. S2B). Both activation of H-Ras and inhibition of p53 increased DNA replication in the MCF10A cells, as demonstrated by the higher proportion of EdU-positive cells (Fig. 2a, b). There were fewer of the modified cells in G1 and more in S and G2/M compared with the parental MCF10A cells (Fig. 2c). They also had higher basal levels of DNA damage, as judged by phosphorylated histone H2AX(γH2AX) staining (Figs. 2d and S2C). These effects were stronger in the MCF10A-HRAS cells than in the MCF10A-GSE56 cells.

FACT depletion in the MCF10A-GSE56 and MCF10A-HRAS cells significantly reduced viability compared with the shControl cells (Fig. 2e). This effect was stronger in the MCF10A-HRAS cells compared with the MCF10A-GSE56 cells, suggesting that the dependence of cells on FACT is elevated proportionally to the degree of replication dysregulation. A significant decrease in the proportion of EdU-positive cells was also observed with the MCF10A-GSE56 and MCF10A-HRAS cells following SSRP1 knockdown, but not with the control MCF10A cells (Fig. 2f). However, there was no increase in caspase-3/7 activity upon FACT depletion in all cell variants (Fig. 2g).

Replication in transformed and tumor cells is frequently accompanied by replication stress. Both overexpression of HRAS^V12^ and loss of p53 function have been shown to induce such stress [28–30]. Indeed, MCF10A-HRAS and MCF10A-GSE56 cells did show signs of increased replication stress, including increased γH2AX staining in basal conditions (Fig. 2d) and an increased occurrence of micronuclei (Fig. 2h). Therefore, we tested whether the induction of replication stress by other means also made MCF10A cells sensitive to FACT knockdown. For these experiments, we used hydroxyurea (HU), which reversibly inhibits ribonucleotide reductase and decreases deoxyribonucleotide biosynthesis, resulting in the inhibition of the progression of DNA replication forks [31]. MCF10A cells expressing shSSRP1 or shControl were treated with 1 mM HU for 12 h and then allowed to grow in the absence of HU. EdU incorporation in HU-treated shControl cells was not significantly different from untreated cells 24 h post-HU treatment. In contrast, HU treatment of FACT-depleted cells caused a significant decrease in EdU incorporation compared with the untreated cells (Fig. 2i). Moreover, there was an ~35% reduction in the long-term viability of FACT-depleted cells compared with shControl cells following HU treatment (Fig. 2j), suggesting that the induction of replication stress with HU sensitized MCF10A cells to FACT depletion similar to the overexpression of mutant HRas or inhibition of p53.

### Loss of FACT increases susceptibility to replication-induced DNA damage in MCF10A cells

We observed that cells with elevated levels of replication stress are more sensitive to FACT depletion, indicating that FACT may be involved in the response to replication stress in mammalian cells. All cells, including normal, non-tumorigenic cells replicating in vitro, experience some level of endogenous replication stress, which can arise from secondary DNA structures, DNA–RNA hybrids, and collisions between replication and transcription complexes [32]. If FACT is critical for protecting cells from replication stress, then we should observe an increase in the sensitivity of MCF10A cells to these endogenous factors.

**Fig. 3:**
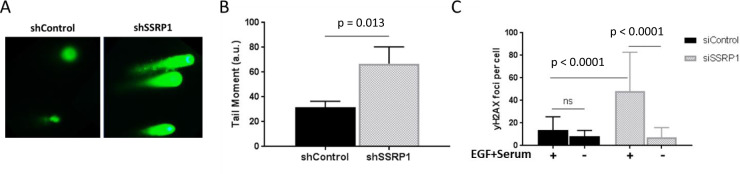
Loss of FACT increases the susceptibility of MCF10A cells to replication-induced DNA damage. **a**, **b** The comet assay was performed under alkaline conditions 72 h post-transduction of MCF10A cells with the indicated lentiviruses. **a** Representative images of the comets. **b** The tail moment of the comets was quantified using ImageJ plugin Open Comet. Data are presented as the mean ± SD (*n* = 6). **c** γ-H2AX staining of MCF10A cells grown in normal medium (+) or EGF- and serum-depleted medium (−) and transfected with the indicated siRNA. γ-H2AX immunofluorescence staining was performed 72 h post-transfection, and the number of foci per cell nucleus was quantified using ImageJ. Data are presented as the mean ± SD (*n* = 3).

The slowed progression through S-phase observed in FACT-depleted MCF10A cells is a strong indicator of increased replication stress. We, therefore, compared the basal level of DNA damage, another marker of replication stress, between control and FACT-depleted MCF10A cells. Although there was no overall decrease in viability or proliferation in the MCF10A cells following FACT depletion, there was an increased tail moment of the comets (Fig. 3a, b) and an increased number of γH2AX foci per cell (Fig. 3c) in the FACT-depleted cells compared with the controls. Importantly, there was no difference in the level of DNA damage between FACT-depleted and control MCF10A cells when cells were growth-arrested in growth factor- or serum-depleted medium (Fig. 3c), confirming that DNA replication is the source of the DNA damage observed in FACT-depleted cells.

### FACT-depleted cells show a loss of intra S-phase checkpoint activation in response to replication stress

**Fig. 4:**
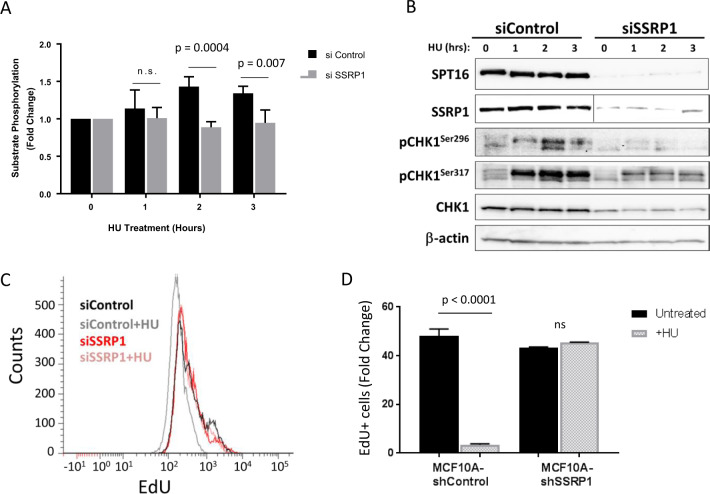
FACT-depleted cells show a loss of ATR/CHK1 activation in response to replication stress. **a** Quantitation of phospho-(Ser/Thr) ATM/ATR substrate levels in lysates from MCF10A cells transfected with the indicated siRNA and incubated in the presence or absence of 2.5 mM HU for the indicated times. The intensities of the four substrate bands of interest were quantified using ImageJ. Bars indicate the mean intensity ± SD. **b** Immunoblotting of MCF10A cell lysates following treatment with 2.5 mM HU for the indicated time points. **c**, **d** EdU incorporation in MCF10A cells 72 h post-transfection with the indicated siRNA and treatment with 2.5 mM HU for 12 h. EdU was added for the last 30 min. **c** Histograms of EdU intensity distribution. **d** Percentage of EdU+ cells. Data are presented as the mean ± SD (*n* = 2).

The major pathway responsible for the detection of replication stress is the ATR/CHK1 pathway [32]. FACT-depleted cells had an increase in the number of micronuclei (Fig. 2g), a marker of genomic instability, suggesting that they may have issues with responding to and overcoming replication stress. To test the ability of FACT-depleted cells to respond to replication stress, we treated MCF10A cells with HU and analyzed ATR/CHK1 pathway activation using a phospho-(Ser/Thr) ATM/ATR substrate antibody that recognizes phosphorylated serine or threonine in the S*/T*Q motif (an essential requirement for ATR substrates) [33]. In control cells, we observed an increase in the number of phosphorylated bands corresponding to the ATR substrates after incubation with HU; however, this increase was lost in FACT-depleted cells (Figs. 4a and S3A). The same effect was observed when we evaluated the phosphorylation of CHK1 on Ser317 and Ser296, two sites phosphorylated by ATR in response to replication stress. FACT depletion decreased the induction of Ser317 phosphorylation and almost completely abrogated Ser296 autophosphorylation in CHK1 in response to HU (Fig. 4b).

One of the consequences of ATR/CHK1 activation in response to replication stress is the inhibition of new origin firing [32]. Thus, we evaluated the replication rate in FACT-depleted cells treated with HU. For this experiment, we treated cells with HU for 12 h and added EdU for the last 30 min of the HU treatment. In the presence of HU, control cells had strongly decreased EdU incorporation; however, there was no change in EdU incorporation in FACT-depleted cells (Fig. 4c, d). These data suggested that FACT-depleted cells are unable to arrest replication under conditions of replication stress. These experiments established that FACT is needed for the activation of the ATR/CHK1 pathway in response to replication stress.

### FACT is necessary for the uncoupling of DNA synthesis and helicase activity, a crucial event for ATR signaling activation

**Fig. 5:**
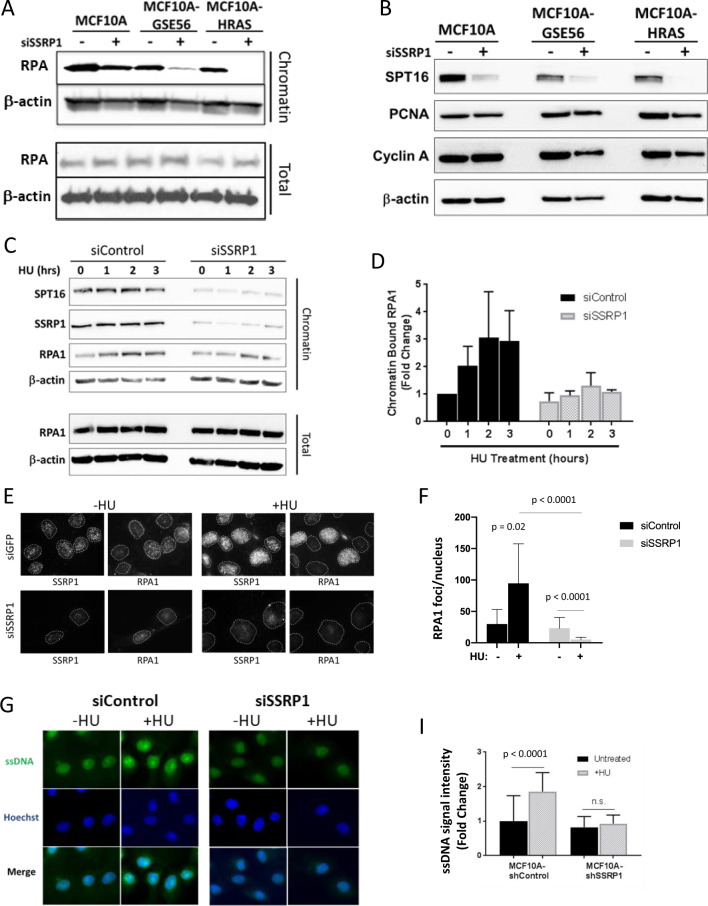
ssDNA does not accumulate upon replication stress in FACT-depleted cells. **a**, **b** Immunoblotting of total and chromatin extracts 72 h after transfection of with siSSRP1 (+) or siControl (−). **c** Immunoblotting of total and chromatin extracts 72 h after transfection of MCF10A cells with siSSRP1 (+) or siControl (−) and treatment with 2.5 mM HU for the indicated time points. **d** Quantitation of panel **c**. Data are presented as the mean ± SD (*n* = 2) for the chromatin-bound RPA1 band intensity relative to total RPA1. **e**, **f** Immunofluorescence staining of MCF10A cells following transfection with either control siRNA (siGFP) or SSRP1 siRNA and treatment with 2.5 mM HU for 6 h. Cells were permeabilized before fixation to remove soluble proteins. **e** Representative images of SSRP1 and RPA1 staining. The contours of cell nuclei used for quantitation are shown with the dotted lines. **f** Quantitation of RPA1 foci per cell. Data are presented as the mean ± SD for 20–50 cells. **g**–**i** Immunofluorescence staining of ssDNA in MCF10A cells following transfection with control (siControl) or SSRP1 siRNA and treatment with 2.5 mM HU for 3 h. **g** Representative images. **i** Quantitation of ssDNA staining per cell presented in **g**. Data are presented as the mean ± SD for 20–50 cells (*n* = 3).

To investigate the mechanism by which FACT helps initiate ATR/CHK1 signaling, we compared the upstream events leading to ATR/CHK1 activation in response to replication stress. When DNA polymerase faces a barrier or does not have enough nucleotides, the helicase continues to unwind the DNA ahead of the stalled DNA polymerase [34]. This activity results in the accumulation of ssDNA, which is recognized and bound by RPA. Accumulation of RPA-bound ssDNA serves as a trigger for ATR activation [34]. We observed a reduced amount of chromatin-bound RPA in all FACT-depleted cells. The reduction in chromatin-bound RPA may be due to the reduced ssDNA levels during replication stress or a reduced number of replicating cells in the population. To select between these two possibilities, we assessed protein levels of two replication markers PCNA and cyclin A and found no change in the total amounts of these proteins as well as RPA (Fig. 5a), suggesting that there is reduction of ssDNA in cells, but not of the proportion of replicating cells. This effect was the strongest in MCF10A-HRAS cells, which was in line with the increase in the basal level of replication stress (Fig. 5a). There was also delayed and reduced accumulation of RPA on chromatin in FACT-depleted MCF10A cells treated with HU compared with the control cells (Fig. 5b–e). We also compared the ssDNA content in control or HU-treated cells using an ssDNA-specific antibody. The control cells had a significant increase in nuclear ssDNA levels upon HU treatment; however, there was no significant change in the amount of ssDNA in FACT-depleted cells treated with HU (Fig. 5f, g). Thus, the absence of FACT not only attenuated ATR signaling but also eliminated the signal for ATR activation (i.e., the emergence of stretches of ssDNA/RPA complexes).

**Fig. 6:**
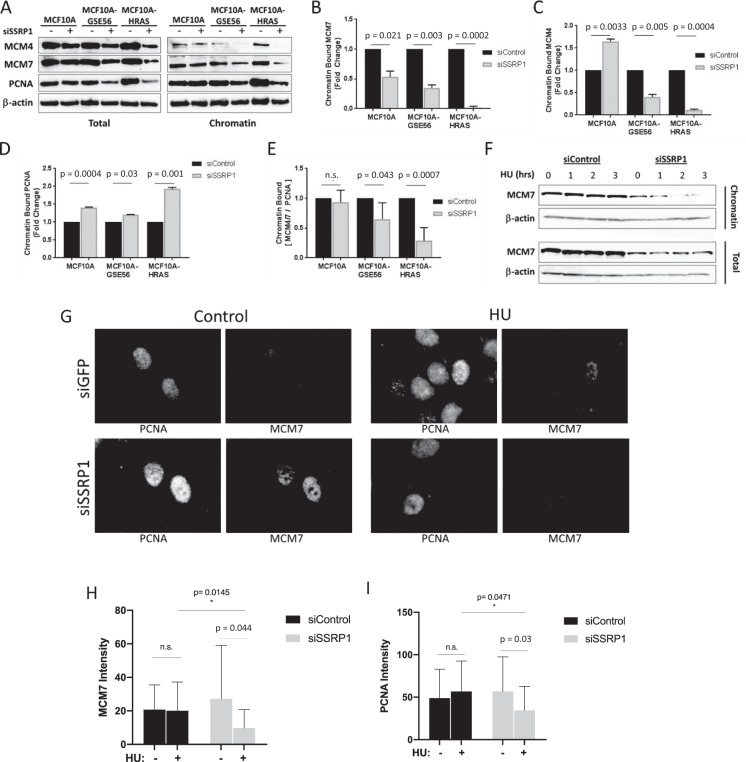
Loss of FACT causes dissociation of the MCM2-7 helicase complex from chromatin during replication stress. **a**–**e** Immunoblotting of total cell and chromatin extracts from cells transfected with the indicated siRNA. **a** Representative blots. The levels of chromatin-bound MCM7 (**b**), MCM4 (**c**), and PCNA (**d**). The band intensities were normalized to β-actin. siControl samples are set equal to 1. **e** Ratio of chromatin-bound MCM4/7 relative to chromatin-bound PCNA. **b**–**e** Mean of two biological replicates ±SD. **f** Immunoblotting of MCF10A cells treated with 2.5 mM HU for the indicated time points. **g**–**i** Immunofluorescent staining of MCF10A cells treated with 2.5 mM of HU for 6 h with washed out soluble proteins before fixation. Quantitation of nuclear fluorescence of 20–50 cells per each condition. Only PCNA-positive nuclei were quantitated for MCM7 (**h**) and PCNA (**i**) signal.

The role of FACT in replication has mainly been attributed to direct interactions with the MCM2-7 helicase complex because FACT was shown to facilitate the unwinding of DNA through chromatin [17, 35]. Moreover, FACT knockdown in transformed DT40 chicken cells leads to decreased fork speed due to the reduced unwinding activity of helicase in the context of chromatin [18]. Therefore, we hypothesized that the interaction of FACT with the MCM2-7 complex may be necessary for the uncoupling of DNA synthesis and helicase activity and the accumulation of ssDNA. To test this hypothesis, we determined what happens to the MCM2-7 helicase in FACT-depleted cells. We measured the levels of total and chromatin-bound MCM4 and MCM7 (two helicase subunits) in MCF10A, MCF10A-GSE56, and MCF10A-HRAS cells transfected with control or SSRP1 siRNAs. In all three cell lines, we saw a significant loss of MCM7 from chromatin with the degree of loss correlating with the sensitivity of cells to FACT knockdown and the basal level of replication stress (Fig. 6a, b). MCM4 was also lost from chromatin upon FACT knockdown, but only in the cells with high levels of replication stress (Fig. 6a, c). To validate that loss of the MCM2-7 helicase from chromatin is not due to a reduced number of cells undergoing replication, we measured the levels of chromatin-bound PCNA, which is a marker of the number of active forks in a cell [36]. We observed an increase in the chromatin-bound PCNA levels upon FACT depletion in all cells (Fig. 6a, d), which indicated a loss of the inhibition of origin firing, because PCNA is only loaded onto chromatin when the origin is fired [36]. Analysis of the fold-change ratio of chromatin-bound MCM4/7 relative to chromatin-bound PCNA demonstrated that this effect represented an MCM2-7 helicase-specific loss from the chromatin (Fig. 6e).

Finally, we monitored MCM7 binding to chromatin in HU-treated cells. While MCM7 binding to chromatin in siControl cells was not changed following treatment with HU for 3 h, MCM7 was gradually lost from chromatin in cells without FACT (Fig. 6f). In contrast, the total amount of MCM7 was not changed irrespective of FACT depletion. We used immunofluorescent staining to confirm these observations. To evaluate only chromatin-bound helicase in cells undergoing replication, we extracted the soluble proteins from the cells with Triton X-100 before fixation. Analysis was also limited to PCNA-positive cells (i.e., cells with ongoing replication). Importantly, we performed controlled co-staining experiments and established that only PCNA-positive cells were positive for MCM7 and RPA70 under these conditions. The intensity of PCNA staining per nucleus showed little change between conditions, except for a slight increase upon FACT loss and a slight decrease following treatment with HU for 6 h. MCM7 was still present on chromatin at 6 h in HU-treated cells with FACT. However, there was a significant reduction in chromatin-bound MCM7 levels in cells without FACT. Thus, in mammalian cells, FACT can prevent MCM2-7 helicase dissociation from chromatin that is induced by replication stress, which is critical for signaling an ATR-dependent replication stress response.

## Discussion

Several recent studies have questioned the role of FACT in facilitating transcription through chromatin as the major reason why FACT is critical for the viability of transformed and tumor cells [2–4, 10]. Indeed, because only proliferating transformed mouse fibroblasts, not growth-arrested cells, are sensitive to FACT loss, a role for FACT in DNA replication may be vital for proliferating tumor cells.

Most previous studies suggested that FACT is required for general replication. Yeast FACT directly interacts with DNA polymerase α [14, 37], and the yeast SSRP1 homolog, Pob3, interacts with the single-stranded DNA-binding protein RPA1 [38], suggesting that yeast FACT is associated with the replisome complex. A direct physical interaction between FACT and the MCM2-7 helicase was also demonstrated in human HeLa tumor cells. This binding was essential for the unwinding activity of the MCM2-7 helicase through chromatin [17, 35]. In yeast and chicken cells, FACT is also loaded together with the MCM2-7 complex at replication origins, and its function is critical for elongation in replication [17, 18, 35]. FACT is also associated with Rtt106 and collaborates with CAF-1 and Rtt106 in replication-coupled nucleosome assembly in yeast cells [39]. Importantly, there is still no clear view on the exact function of FACT during replication (e.g., suggested roles in nucleosome disassembly, nucleosome assembly at newly synthesized DNA, prevention of histone loss, potential nucleosome-independent functions).

Intriguingly, our lab and others have demonstrated ongoing replication in non-tumor cells with little or no FACT [4, 21, 40]. Moreover, Mylonas and Tessarz [7] showed that FACT knockdown increased the proliferation of mouse ESC. To explain these differential effects of FACT depletion on DNA replication in mammalian cells, we proposed that FACT is required for replication in cells with high levels of replication stress. Our data demonstrated that FACT is needed for the association of the MCM2-7 helicase with chromatin, but only when it is uncoupled from DNA polymerase during replication stress. This uncoupling is a critical event that enables cells to sense and respond to replication stress [34]. Our data suggest that FACT stabilizes the MCM2-7 complex on chromatin when DNA polymerase is left behind. Without FACT, the helicase is rapidly dissociated from chromatin without the generation of the stretches of ssDNA needed for the activation of the ATR/CHK1 response to replication stress.

It is unclear how FACT helps the MCM2-7 helicase remain on chromatin and facilitates its unwinding activity. The most straightforward hypothesis is that FACT allows the helicase to move through the nucleosome via its nucleosome disassembly activity. However, there are several problems with this explanation. First, this answer would suggest that FACT is needed for general replication in all eukaryotic cells, including non-tumor mammalian cells. Second, there is increasing evidence that mammalian FACT does not have actual nucleosome disassembly activity (i.e., it cannot bind fully assembled nucleosome wrapped with DNA) [41–43]. At least in cell-free conditions, MCM2-7 alone cannot unwind nucleosomal DNA [44]. Thus, the more probable scenario is that cooperation between FACT and the MCM2-7 complex is needed for this process. When the MCM2-7 helicase approaches a nucleosome, it may cause partial unwrapping of DNA from the core, which then allows FACT to bind to histone oligomers and release additional fragments of DNA for helicase-mediated unwinding. The problem with this model is that FACT cannot compete with DNA for histone binding [45]. However, the previous experiments were done with double-stranded DNA [17, 35]. ssDNA generated by the MCM2-7 helicase and potentially RPA-coated ssDNA may be less potent competitors with FACT for histone core binding. The testing of this model will require structural studies.

Alternatively, it is known that the progress of the replication fork depends on nucleosome assembly behind the fork (reviewed in [46]). The mechanism of this dependence has not been established but may be related to interference of fork progression by the free histones removed from nucleosomes due to their irregular binding to the DNA ahead of the fork. These histones must be transferred from ahead of the fork to behind the fork, which is a function ideally suited to FACT because it can bind all nucleosome components and tether them together. Blocking nucleosome assembly slows down replication and prevents DNA polymerase MCM2-7 uncoupling and ssDNA accumulation in response to HU-induced replication stress [47]. In fact, this effect was observed upon knockdown of histone chaperone CAF-1 [48, 49] and by short-term deprivation of the histone supply [47].

FACT has previously been implicated in replication stress. Yeast with mutant Pob3 (homolog of SSRP1) [15, 50] or lacking the N-terminal domain of SPT16 were very sensitive to HU-induced replication stress [50]. In addition, FACT depletion from HeLa and MRC-5 (normal embryonic human fibroblasts) cells increased DNA damage and R-loop formation [51]. However, these cells were rescued from DNA damage by RNase H1 overexpression, suggesting that these cells experienced transcription-dependent replication stress upon FACT depletion [51].

The increase in DNA damage upon FACT depletion in cells is most likely due to the destabilization and collapse of replication forks that are unable to overcome certain types of replication stress, as suggested by the loss of the MCM2-7 complex from chromatin upon FACT knockdown. MCF10A cells have low basal levels of endogenous replication stress and functional DNA repair and checkpoint pathways [22]. Thus, these cells are viable and can proliferate with only minor consequences in the absence of FACT. When challenged with higher levels of replication stress, these cells lose their ability to replicate in the absence of FACT. Tumor cells that have persistent replication stress are unable to activate the ATR/CHK1 checkpoint response in the absence of FACT. Therefore, they cannot stabilize the replication forks and accumulate DNA damage and die. Our study demonstrates a role for FACT in protecting the integrity of DNA replication under conditions of replication stress. This function is not critical in non-tumor cells with low levels of endogenous replication stress but becomes essential upon oncogene activation, loss of tumor suppressors, or exogenous sources of replication stress. Most previous studies on FACT function in DNA replication used cells with high endogenous levels of replication stress (e.g., human tumor or chicken lymphoma cells) [18]. High basal levels of replication stress were also observed in yeast compared with normal mammalian cells [52, 53].

Expression of oncogenes and loss of efficient cell cycle checkpoint signaling induce high levels of replication stress during the early stages of tumor development [54]. Factors, such as histone chaperones and other chromatin modifiers, may also become dysregulated to help alleviate or protect cells from this increase in replication stress and promote cancer development. Another histone chaperone, ASF1, has already been proposed to play such a role [55, 56]. We have demonstrated that FACT upregulation is critical during oncogene-induced transformation. Without this upregulation, mammary epithelial cells succumb to oncogene-induced senescence, a major consequence of unmanageable replication stress in premalignant cells. Moreover, FACT expression is essential for maintaining the viability and proliferation of breast cancer cells [3].

The abrogation of the ATR/CHK1 replication stress response checkpoint in the absence of FACT suggests that FACT inhibition could be used to target DNA replication in cells with preexisting high levels of replication stress (e.g., cancer cells) with minimal effects on cells with low levels of replication stress (i.e., normal cells). Direct ATR inhibitors have shown strong anticancer activity and are currently being developed and tested in clinical trials [20]. However, the ATR pathway plays other roles in addition to the replication stress response (e.g., DNA damage response). Moreover, many ATR inhibitors also inhibit ATM, which can lead to toxicity in normal cells, especially in combination with DNA damaging chemotherapeutic agents [20]. In this study, we showed that loss of FACT leads to the inhibition of the ATR response to replication stress, presumably without interfering with the replication stress-independent functions of ATR. Thus, targeting FACT could represent a therapeutic strategy to sensitize tumor cells to their high levels of replication stress. The success of such therapy will depend on whether or not FACT inhibition is toxic to any normal cell types in mammals, which is currently under investigation.

## Methods

### Cells

MCF7 and MCF10A cells were obtained from ATCC (Manassas, VA). All cells used in this study were maintained at 37 °C and 5% CO_2_. MCF7 cells were grown in DMEM (Invitrogen) supplemented with 10% FBS (Invitrogen, Carlsbad, CA) and 100 U/ml penicillin/streptomycin (Invitrogen). Cells were routinely tested for mycoplasma. MCF7 cells were authenticated by STR (100%). MCF10A, MCF10A-GSE56, and MCF10A-HRAS^V12^ cells were grown in DMEM/F-12 supplemented with 5% horse serum (Invitrogen), 10 μg/ml insulin (Invitrogen), 5 μg/ml hydrocortisone (Sigma-Aldrich, St. Louis, MO), 20 ng/ml epidermal growth factor (Sigma-Aldrich), 100 ng/ml cholera toxin (MilliporeSigma, Burlington, MA), and 100 U/ml penicillin/streptomycin (Invitrogen). MCF10A-HRAS^V12^ cells were generated as previously described [4]. MCF10A-GSE56 cells were generated by transfection of MCF10A cells with pLX-Neo-GSE56 using lipofectamine 2000 (Invitrogen), followed by selection with 500 μg/ml neomycin (G418 Sulfate).

Mouse skin fibroblasts from *Ssrp1*^*fl/fl*^/CreER^T2+/+^ mice were transformed in culture with GSE56 and HRAS^V12^, as previously described [8, 21]. The cells were plated at high density (~100% confluency) or low density (~10% confluency) and treated with 2 µM 4-OHT (Sigma-Aldrich) or vehicle for 120 h. Low-density and high-density cells were grown in DMEM containing 100 U/ml penicillin/streptomycin and 10% or 0.5% FBS, respectively. The medium was replaced every 48 h with fresh 2 µM 4-OHT.

### Antibodies and reagents

The following antibodies were used for immunoblotting, immunofluorescence, or flow cytometry: SSRP1 (609702; Biolegend, San Diego, CA), SPT16 (607002; Biolegend), HRAS (sc-520; Santa Cruz, Dallas TX), p53 (ab27696, Abcam, Cambridge, United Kingdom), p21 (sc-6246; Santa Cruz), γH2AX (9718; Cell Signaling, Danvers, MA), RPA70 (2267; Cell Signaling), MCM7 (sc-9966; Santa Cruz), MCM4 (ab4459; Abcam), phospho-(Ser/Thr) ATM/ATR substrate antibody (2851; Cell Signaling), Chk1 (2360, Cell Signaling), p-Chk1-Ser317 (2344; Cell Signaling), p-Chk1-Ser296 (90178; Cell Signaling), cyclin A (4656; Cell Signaling), PCNA (2586; Cell Signaling), ssDNA (MAB3868; MilliporeSigma), Cross-Adsorbed Secondary Antibody, Alexa Fluor 488 (A-11008; Thermo Fisher Scientific, Waltham, MA), and β-actin (A3854, Sigma-Aldrich). Where indicated, cells were treated with 1 or 2.5 mM HU (H8627; Sigma-Aldrich). Cell cycle analysis was done using propidium iodide or Hoechst 33342 stain. CBL0137 (1 μM; Incuron, Buffalo, NY, USA) was used as a positive control for p53 activation and p21 induction.

### RNA interference

SSRP1 was knocked down using both siRNA and shRNA against SSRP1. In some experiments, SUPT16H shRNA was used. MISSION^®^ esiRNA targeting human SSRP1 or SUPT16H was obtained from Sigma-Aldrich. Transfection of siSSRP1 was performed with Lipofectamine 2000 reagent (11668-027; Invitrogen), and assays were performed 72 h post-transfection. shRNAs to the 3′-UTR of SSRP1 and control shRNA (scrambled) were synthesized and cloned into a lentiviral vector by GeneCopoeia (Rockville, MD, USA). The target sequences were gtccctggattctgtgcca (shSSRP1-1) and cagtggggagacgtctta (shSSRP1-2). Lentiviral packaging was performed as previously described (4). The virus was concentrated using Amicon Ultra-4 centrifugal filter units (MilliporeSigma).

### Cell viability assay

Cell viability was measured using the methylene blue assay. Cells were grown for the indicated times, and the media aspirated. The cells were washed with PBS and fixed and stained with 0.5% methylene blue (M4159; Sigma-Aldrich) in 50% methanol for 30 min at room temperature. The plates were washed with water, air dried, and photographed. The dye was eluted with 1% SDS and quantified using the PerkinElmer^®^ Multimode VICTOR^™^ X3 plate reader (PerkinElmer, Waltham, MA, USA). All assays were repeated at least twice.

### Western blotting

Total cell lysates were prepared using 1× Cell Culture Lysis Reagent (CCLR) (E1531; Promega, Madison, WI) supplemented with protease inhibitor cocktail and PhosSTOP^™^ phosphatase inhibitor (Sigma-Aldrich). Lysates were incubated on ice for 30 min with vortexing every 10 min, followed by sonication twice for 5 min using the Biorupter^™^ UCD-200 (Diagenode, Denville, NJ, USA). Soluble protein was collected by centrifugation of the total cell lysates at 10,000 rpm for 10 min at 4 °C. Insoluble (chromatin-bound) protein was obtained by resuspension of the remaining pellet in 1× CCLR, followed by sonication. The protein concentration was performed using the Pierce^™^ BCA Protein Assay (23225; Invitrogen). Western blotting was performed using standard method. Bands were visualized using Western Lightning Plus-ECL, Enhanced Chemiluminescence Substrate (NEL103E001EA, PerkinElmer, Waltham, MA) on the FluorChem H2 scanner (ProteinSimple, San Jose, California, USA) and quantified using ImageJ software. Data were normalized to β-actin. Where indicated, the percentage of chromatin-bound protein was calculated by finding the ratio of normalized chromatin-bound protein to total protein relative to the control cells. Experiments were run at least twice with use of two technical replicates (two plates of cells, processed simultaneously and identically).

### Apoptosis assay

To measure apoptosis in FACT-depleted cells, the medium was removed from cells plated in 96-well plates, and 20 μl of lysis buffer (50 mM HEPES, 0.1% CHAPS, 2 mM dithiothreitol, 0.1% Nonidet P-40, 1 mM EDTA, and 1% protease inhibitor) and 20 μl of caspase assay buffer (100 mM HEPES, 10% sucrose, 0.1% CHAPS, 1 mM EDTA, 2 mM dithiothreitol, and 50 μM caspase-3/7 substrate ((Ac-DEVD-AMC) Cat# ALX-260-031-M005; Enzo Life Sciences, Farmingdale, NY) were added. Basal activity was measured at an excitation wavelength of 380 nm and an emission wavelength range of 430–460 nm using a spectrofluorometer. The cells were then incubated at 37 °C, and caspase-3/7 activity was measured 5 and 24 h after the basal reading. Experiment was repeated two times.

### Click-IT EdU assay

Cells were incubated with 20 μM EdU for designated times. EdU incorporation was measured using the Click-iT^®^ EdU Alexa Fluor^®^ 488 Imaging and Click-iT^®^ Plus EdU Alexa Fluor^®^ 647 Flow Cytometry Assay kits (Invitrogen), according to manufacturer’s protocol. Experiment was run more than two times.

### Immunofluorescence

Cells were fixed with 4% paraformaldehyde for 20 min and permeabilized and blocked in PBS containing 0.1% Triton X-100 and 1% BSA for 30 min. For assessment of chromatin-bound proteins, cells were incubated with 1% Triton X-100 for 10 min at room temperature, fixed with cold methanol for 5 min, and washed with PBS before blocking. Cells were incubated with primary antibody for 1 h in permeabilization buffer. Fluorescence microscopy was performed using the Axio Observer A1 inverted fluorescence microscope and AxioVision software (Carl Zeiss AG, Oberkochen, Germany). The intensity of fluorescence staining or foci per cell was collected at least from 20 cells (in many cases from 50) per condition and quantified using ImageJ. All experiments were repeated two times.

### Comet assay

The comet assays were performed using the OxiSelect^™^ 96-Well Comet Assay kit (cat# STA-355; Cell Biolabs, San Diego, CA) in alkali conditions, according to the manufacturer’s protocol. The tail moment was quantified using the Open Comet plugin for ImageJ.

### Flow cytometry

Flow cytometry was performed using the LSR Fortessa A and BD LSRII UV A cytometers (BD Biosciences, San Jose, CA, USA), and the data were analyzed using WinList 3D (Verity Software House, Topsham, ME, USA). The fluorophore intensities were measured using the area. The graphs were generated using WinList^™^ 3D 7.1 and FCS Express 4.

### Statistical analysis

Data were compared between the control and treated groups using the Mann–Whitney test. Analyses were conducted using GraphPad Prism 7.03 software.

## Supplementary information

SUPPLEMENTAL MATERIAL

## Data Availability

All materials are available upon request.
