# Supplementary material for: Histone chaperone FACT is essential to overcome replication stress in mammalian cells
Source: Oncogene. 2020 Jun 12;39(28):5124–37. doi: 10.1038/s41388-020-1346-9 (PMC7343669; doi:10.1038/s41388-020-1346-9)
Supplement: Supplementary file 1 — SUPPLEMENTAL MATERIAL [file 41388_2020_1346_MOESM1_ESM.pdf]

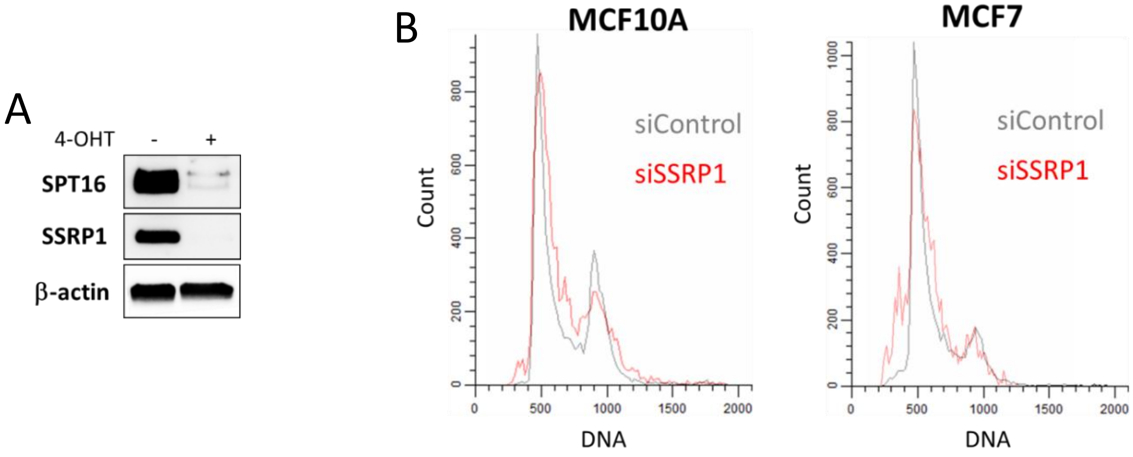

Figure S1. (A) Effect of 4-OHT treatment on FACT levels in mouse transformed fibroblasts from *Ssrp1*$^{fl/fl}$;*CreER*$^{T2+/+}$ mice. Immunoblotting of lysates of cells treated with 4-OHT for 5 days. (B) Cell cycle analysis of FACT depleted MCF10A (left) and MCF7 (right). 72 hours after transfection with siControl or siSSRP1 cells were collected and fixed for flow cytometry. PI area was used to determine DNA content on x-axis.

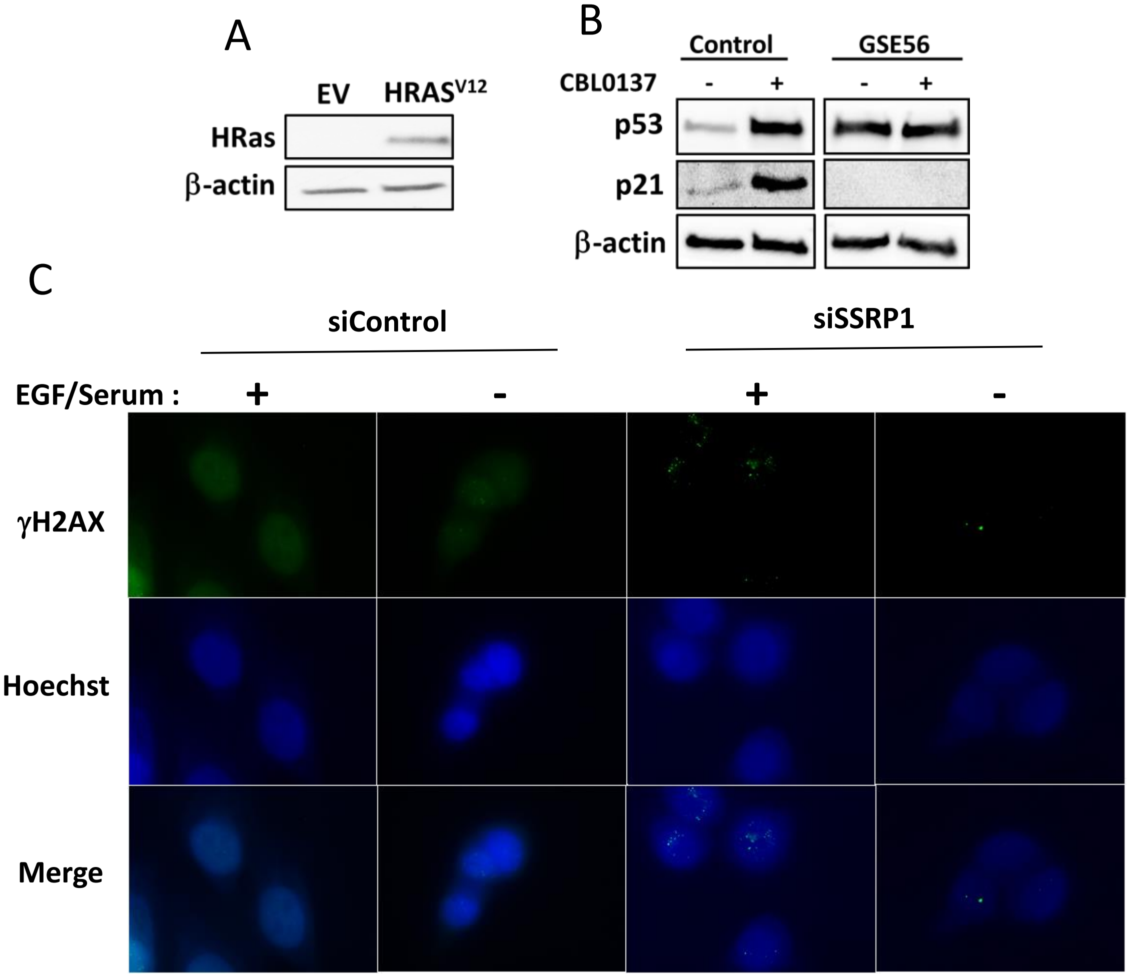

Figure S2. (A) Immunoblotting of MCF10A cells transduced with empty lentivirus (EV) or harboring expression construct for $HRAS^{V12}$ after two weeks selection on bleomycin. (B) Testing of functional status of p53 in control MCF10A cells and cells expressing GSE56. Immunoblotting of lysates of cells treated with 1uM of CBL0137 for 16 hrs. (C) γH2AX Immunofluorescence images of MCF10A cells transfected with the indicated siRNAs and maintained either in normal growth medium (with serum and EGF) or in serum and EGF deficient medium.

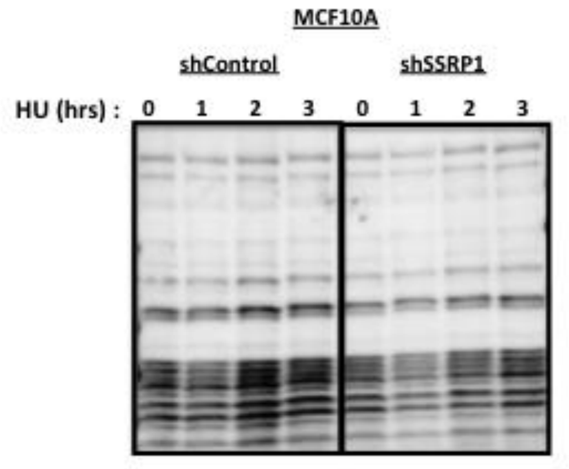

Figure S3. ATM/ATR activation in response to replication stress caused by HU in MCF10A cells transduced with lentiviruses with indicated shRNAs. Immunoblotting of lysates of cells with antibody to ATM/ATR phosphorylation substrates.
